# Thematic Analysis of Clinician Documentation Surrounding Treatment Discussions and Decision‐Making in Patients With Desmoid Tumors

**DOI:** 10.1002/cam4.71183

**Published:** 2025-08-23

**Authors:** Victoria Wytiaz, Tianyi Wang, Scott Schuetze, Nina J. Francis‐Levin, Rashmi Chugh

**Affiliations:** ^1^ Division of Oncology, Department of Internal Medicine University of Michigan Ann Arbor Michigan USA; ^2^ School of Medicine University of Michigan Ann Arbor Michigan USA; ^3^ Division of Metabolism, Endocrinology and Diabetes, Department of Anthropology University of Michigan Ann Arbor Michigan USA

**Keywords:** aggressive fibromatosis, mental health, neoplasms, psychosocial distress, therapeutics

## Abstract

**Background:**

Desmoid tumors are benign tumors without metastatic potential. While progression is rarely life‐threatening, it is often life‐affecting with significant morbidity. The rarity of desmoid tumors and the inability to accurately predict their natural history lead to challenges in developing treatment algorithms or formulaic discussions to address treatment options with patients.

**Aims:**

To better understand this process, we performed a qualitative study of clinician documentation for patients with desmoid tumors to identify themes and areas for improvement.

**Methods:**

An institutional database search was used to identify patients with desmoid tumors seen from 2018 to 2023. Documentation entries in the electronic health record (EHR) were reviewed, and passages pertaining to treatment were abstracted and explored using inductive thematic analysis.

**Results:**

Ninety‐five patients were identified. Generated themes included (1) balancing uncertainty and ambiguity with hope, (2) empowerment through patient‐initiated treatment discussions, (3) maintaining an active lifestyle and mitigating psychosocial distress, and (4) fostering longitudinal clinician–patient relationships and building trust.

**Conclusions:**

The current analysis of documentation pertaining to treatment for patients with desmoid tumors provides insight into clinician–patient discussions and decisions in a real‐world clinical setting. The emergent themes provide novel guidance and support for optimizing treatment discussions and decisions. More robust community peer support groups specific to patients with desmoid tumors and clinician “support groups” may provide a way for both patients and clinicians practicing in a disease state with uncertainty to share experiences and improve resilience. Developing treatment educational materials for patients and families and improving access to supportive psychosocial services may further enhance high‐quality decision‐making.

## Graphical abstract

Discussions about treatment for patients with desmoid tumors have themes that include balancing uncertainty and ambiguity with hope, empowerment through patient‐initiated treatment discussions, maintaining an active lifestyle and mitigating psychosocial distress, and fostering longitudinal clinician–patient relationships and building trust. This analysis of documentation pertaining to treatment for patients with desmoid tumors provides insight into clinician–patient discussions and decisions in a real‐world clinical setting.

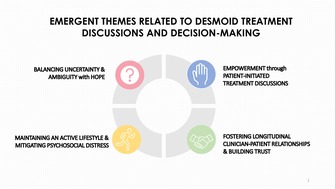

## 1 Background

Desmoid tumors are locally aggressive tumors without metastatic potential [1]. However, these tumors have a variable natural history, and while progression is rarely life‐threatening, it is often life‐affecting with significant morbidity [2]. Desmoid tumors frequently affect young adults during an active and productive portion of their lives when hindrances from illness or treatment toxicities can be particularly detrimental [3]. Treatment options range from expectant management for asymptomatic, stable disease to local measures (surgery, radiation therapy, and cryoablation) and systemic therapy for advanced or progressive disease.

In addition to physical limitations caused by desmoid tumors or their treatments, high levels of emotional distress have been reported in patients with desmoid tumors undergoing systemic therapy or participating in expectant management [4, 5]. There are also challenges for patients in understanding desmoid tumors and their unpredictable but nonmalignant course, especially as most patients receive care at a cancer center [6]. This leads to difficulty for patients in explaining their diagnosis and treatment decisions to peers or family members and ultimately gaining the social support network seen in other malignant tumors [7].

Developing treatment algorithms or formulaic discussions to address treatment options for an individual patient is complex. The rarity of desmoid tumors contributes to the challenges in treatment discussions as objective data (relating to response rates, etc.) may be less readily available or pertinent [8]. The process by which clinicians describe and present treatment options for patients with desmoid tumors is largely uncharacterized. A more comprehensive review of the nature of these discussions surrounding desmoid tumor‐related treatment options and decision making may identify areas for improved clarity to ultimately facilitate patient understanding of a unique disease and to alleviate patient emotional distress.

To better understand the current state of discussions of treatment in patients with desmoid tumors, we performed a qualitative study of clinician documentation in the electronic health records (EHRs) of patients with desmoid tumors referred to a tertiary cancer center. We used reflexive thematic analysis to review the documentation and generate themes with a goal of identifying the current processes of treatment discussions and potential areas for improvement in a complex space.

## 2 Methods

Using a previously published approach, we performed a qualitative study of clinician documentation in patient EHRs [9, 10]. An institutional database search was used to identify patients with desmoid tumors seen from 2018 to 2023 using the key terms “desmoid tumor” or “desmoid fibromatosis” and “treatment.” This search provided 324 unique patients with both terms utilized at any time in the EHR during the 5‐year period. A review and a text search of the individual EHR for each patient was performed for these same terms. Documentation was reviewed, and the following groups were excluded: (1) patients with no documented diagnosis of desmoid tumor; (2) patients for whom the term “treatment” was documented but with no evidence of discussion; and (3) patients for whom the term “treatment” was related to another diagnosis. We reviewed the EHR of this final cohort (*n* = 95) and abstracted a total of 195 passages pertaining to treatment for desmoid tumors (135 medical oncology progress notes, 14 surgical oncology progress notes, 25 telephone calls, 7 tumor board notes, 6 patient messages, 5 pharmacy notes and 3 social work notes).

Passages were explored in Microsoft Word by the first author (VW, medical oncologist) using reflexive thematic analysis, an approach for analyzing unstructured text to facilitate the discovery of previously unidentified factors associated with a phenomenon of interest [11, 12, 13, 14]. Reflexive thematic analysis is considered a theoretically flexible method for developing, analyzing, and interpreting patterns across a qualitative dataset [11, 12]. In reflexive thematic analysis, the researcher's position as an inherently subjective party is noted, and multiple coders are not deemed necessary to avoid bias, as is the case in coding reliability and codebook thematic analyses [15, 16]. This stems from the principle that different coders will notice and make sense of data in different ways and that coding can never be truly accurate. The approach to coding was one modeled on latent content analysis in which interpretation of EHR documentation was undertaken to create implied meaning from both the patient and provider experience [17, 18]. This approach to coding does acknowledge that the study team is inherently involved in the process and that, as such, distance from the object of study is impossible. For this study, an exploratory one in nature and not previously undertaken to our knowledge, we valued the flexibility of reflexive thematic analysis and valued the role of the researcher as a clinician as a strength. Authors SS and RC, also cancer clinicians, served the role of “critical friends” in that their review of the emergent themes challenged the first author (VW) to consider their construction of themes and explore alternative interpretations that resonated with their professional experiences [19]. In addition, full excerpts from EHR documentation are included to provide support for the proposed themes, as practices such as inter‐coder reliability to ensure accuracy are not consistent with the goals of reflexive thematic analysis.

The steps of reflexive thematic analysis include data familiarization, initial coding, generation of initial themes, developing and reviewing themes, refining and defining themes, and finalization of the report [11, 12, 13]. The concept of “information power” was utilized to determine that for this exploratory study, the passages identified yielded an appropriate richness of data and as such, no further patient records or passages were sought [20, 21]. This study was approved by the institution's Institutional Review Board (IRB), approval #HUM00252157. Informed consent requirement was waived given the use of existing data for secondary analysis.

## 3 Results

Ninety‐five patients were identified for further analysis, (65 females and 30 males), with a median age of 35 years at the time of diagnosis (range 14–76). Years of follow‐up ranged from less than 1 year to over 13 years. 78 patients (82%) were white. The location of desmoid tumors varied with intra‐abdominal or mesentery tumors, extremity tumors, and chest/chest wall tumors representing the majority (41%, 25% and 16%, respectively). 73 patients (78%) received at least one type of systemic therapy for their desmoid tumor, while 24 patients (25%) and 17 patients (18%) underwent surgery or another type of local therapy. Demographic information is shown in Table 1. Documentation was extracted from the EHR and included progress notes associated with a clinical encounter, summaries of telephone encounters with clinicians, and written messages from patients and clinicians sent via the EHR system. Clinicians included medical oncologists, surgical oncologists, advanced practice providers (physician assistants and nurse practitioners), and nurses.

**TABLE 1:** Demographic and desmoid tumor‐related characteristics.

	*N* (95)	%
Median age (years) at diagnosis (range)	35 (14–76)	
Gender		
Female	65	68
Male	30	32
Race		
White	78	82
Black	5	5
Hispanic	5	5
Middle Eastern	5	5
Asian	2	3
Desmoid tumor location		
Abdominal/mesentery	39	41
Extremity	24	25
Chest/chest wall	15	16
Head/neck	6	6
Other	11	12
Diagnosis of FAP (familial adenomatous polyposis)	11	12
Surgery at any time	24	25
Systemic therapy at any time	73	77
Other local therapy at any time	17	18

**FIGURE 1:**
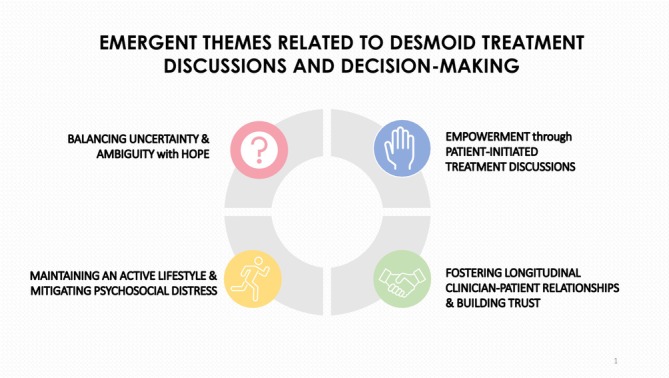
Emergent themes.

In this qualitative analysis of EHR documentation related to desmoid treatment discussions and decision‐making, four themes were generated: (1) balancing uncertainty and ambiguity with hope, (2) empowerment through patient‐initiated treatment discussions, (3) maintaining an active lifestyle and mitigating psychosocial distress, and (4) longitudinal clinician–patient relationships and building trust. See Figure 1.

1. Balancing Uncertainty and Ambiguity With Hope

Documentation referenced uncertainty and ambiguity inherent in the natural history and treatment of desmoid tumors (additional supporting quotations in Table 2). A progress note from a medical oncologist discussing treatment options with a young adult patient and their partner stated that:

The patient and his wife had questions about ideal timing of treatment for desmoid tumors. I discussed that this is fairly subjective and if there is evidence of growth/impending problems, we may wish to start therapy.

Uncertainty was also reflected in determining the appropriate time to discontinue therapy as another medical oncologist shares the lack of data to guide this decision in a response to a patient's messaged question:

There is no right answer. If you feel unwell on the sorafenib [oral medication], you can stop taking it, since it has been 6 months, and you have had response. However, we generally recommend taking it for 9–12 months or until best response is achieved, then moving to surveillance. There is no data to suggest taking it for 3–6 more months will cause additional harm.

This lack of clarity and its resultant impact on patient anxiety was appreciated by clinicians. A patient expressed anxiety to her medical oncologist who documented their impression in the progress note: “Overall, the patient has been quite anxious about her scans. She is very tearful as she is concerned that there was bad news, and she would need to resume treatment.”

**TABLE 2:** Example quotations illustrating the themes of uncertainty and ambiguity & patient‐initiated treatment discussions.

Source	Example quotations: balancing uncertainty and ambiguity with hope
Medical Oncology Progress Note	*He is overall tolerating treatment well, but he does not seem to have a significant change. He does seem to be handling his wound okay without complication from the sorafenib. Is difficult to know how much impact the sorafenib is making and if tumor shrinkage will occur*
Medical Oncology Progress Note	*Although there has not been size decrease in his tumor, he is having improved symptoms related to the tumor since starting sorafenib. It is difficult to know if the symptoms would recur when he stops the medication, however we did review that it is always his choice whether or not to continue*
Medical Oncology Telephone Note	*I am hopeful that the next treatment we try for her desmoid will be tolerated well and help her pain but in the meantime, she may need some increased medications to help with her symptoms*
Medical Oncology Progress Note	*I discussed with her that there is not clear data that more cycles would ensure a longer remission however is certainly possible that the tumor might start to act up sooner with last treatment*
Surgical Oncology Progress Note	*Extensive discussion held regarding treatment options and how there can all have variable success. We opted to observe at this time and discuss at tumor board to see if there are options*
**Source**	**Example Quotations: Empowerment Through Patient‐Initiated Treatment Discussions**
Patient EHR Message	*Beyond the bruising, the increased growth convinces me that I should do some kind of treatment sooner rather than later. I would love to discuss the new trial coming or other treatments that you'd recommend*
Medical Oncology Progress Note	*The desmoids have been stable for a number of years and we discussed she does not need yearly evaluation but can return to clinic for evaluation is she notices increase size or onset of discomfort*
Patient EHR Message	*I'm concerned that the changes in sensation and mobility near my knee are progressing too quickly to wait until December for treatment*
Medical Oncology Progress Note	*She does report less tolerance for the menstrual/hormonal changes she attributes to being on drug and is considering whether or not it might be time for a treatment break soon as she is coming up on 3 years of treatment*
Surgical Oncology Progress Note	*Discussed option to spread out imaging and switch to MRIs. Patient not interested in MRI but comfortable in stretching out surveillance*

The challenge of interpreting desmoid‐related symptoms was documented in a progress note discussion of potential pain from a desmoid tumor:

There is no test to determine if pain is related to the desmoid, but tumors can cause pain out of proportion to the lesion. In order to determine if pain is related to the desmoid, she would need to restart treatment to see if the pain improves.

Finally, within the theme of ambiguity and uncertainty, a representation of hope was also evident. In the face of uncertainty, there is the possibility of a negative, undesirable outcome as well as a positive, desirable one. This latter possibility, or hope, was evident in one medical oncologist's progress note proposing a discontinuation of therapy for a patient:

I discussed that at this point, it's reasonable to initiate a chemotherapy holiday. The effects of treatment can be long‐lasting, and I am hopeful he can remain off therapy for some time, if not indefinitely.

Hope was also shared in another medical oncologist's progress note when modifying therapies to achieve a balance of efficacy and tolerance for their patient:

She is nervous about restarting this medication as she feels so much better after discontinuing. I am hoping that decreasing the dose will be helpful.

2 Empowerment Through Patient‐Initiated Treatment Discussions

Discussions about desmoid‐directed treatment were frequently initiated by patients or family members (additional supporting quotations in Table 2). The impetus for these discussions may have been from sources other than the primary oncology clinician. One patient shared her thoughts on a potential new treatment option with her medical oncologist via EHR message:

Based on what I read in the articles, my main questions are about managing side effects as I still want to work during treatment. I also prefer to try nirogacestat [oral medication] since the drug seems to work quickly. If it's possible to have an appointment, even over the phone, that would be great.

Another patient requested additional educational material regarding the proposed treatment for his desmoid tumor. This was documented by a medical oncology nurse in a phone encounter:

I spoke with patient and reviewed recommendations. I will send him information about liposomal doxorubicin in the portal. He would like to do some research on his own before deciding on a treatment plan.

Patients demonstrated their level of knowledge surrounding treatment options in communication with other clinicians. One patient shared the following in an EHR message to her primary care physician:

I am going try celecoxib [oral medication], a low‐risk treatment for desmoids. While it is not necessarily super‐effective, there's really no treatment that shows overwhelming effectiveness and ones that are more effective have worse side effects.

Family members were frequently involved in treatment discussions. One young patient's father shared his belief that further treatment discussions were warranted in order to prevent future complications as his surgical oncologist notes:

Patient's father expresses concern about if surgical intervention should be done now. He is worried about a surgery being more difficult if the desmoid tumor continues to grow.

A patient experiencing mild side effects from desmoid‐directed therapy shared these symptoms and her decision with acceptance from her medical oncologist who notes:

The patient endorses upset stomach today but shares this may be related to medication or anxiety. She will continue medication for now but would consider stopping if the benefit doesn't outweigh the risk, which is reasonable.

In a similar way, a follow‐up phone conversation with a medical oncology nurse demonstrated a trend in deference to patients as the primary decision‐maker:

How are you feeling? Do you have a decision about if you would like to restart the chemotherapy at lower doses or if you would like to have a treatment break now and consider restarting in the future?

In one scenario, the medical oncologist documented comfort with a patient‐led treatment decision that slightly deviated from the clinician recommendation:

The patient has now been on sorafenib for about 2 years. He stopped treatment 2 weeks ago and noticed slight increase in symptoms. It is reasonable to continue, but after discussion, he would like a trial off of medication to see if symptoms are tolerable.

3 Maintaining an Active Lifestyle and Mitigating Psychosocial Distress

Minimizing disruptions to an active lifestyle was a priority in desmoid treatment‐related discussions (additional supporting quotations in Table 3). One patient shared his desire to avoid altering his day‐to‐day routine as his medical oncologist documents: “He is wondering how treatment for this disease will fit into his life and has questions about treatments closer to home.”

**TABLE 3:** Example quotations illustrating the themes of improvement of quality of life and distress mitigation & longitudinal clinician–patient relationships and building trust.

Source	Example quotations: maintaining an active lifestyle and mitigating psychosocial distress
Medical Oncology Progress Note	*He is wondering how treatment for this disease will fit into his life and has many questions today about local treatments and treatments if he moves distantly*
Medical Oncology Progress Note	*Adding tamoxifen to current treatment regimen given limited side‐effect profile that would likely not be invasive to quality of life. Discussed however that there is possibly an equal likelihood of tumor regression without tamoxifen*
Response to Patient EHR Message	*She is wondering about feasibility of conception, as well as her ability to carry a pregnancy to term in the setting of her tumor*
Medical Oncology Progress Note	*Overall, the patient is grateful for the benefit she is seen with sorafenib in that she can tolerate it but is also upset about the ongoing effects on her life*
Medical Oncology Progress Note	*Patient is inclined to be more aggressive with treatment and favors sorafenib as she is hoping for shrinkage resulting in the tumor having less impact on her ability to do certain exercises when working out*
**Source**	**Example Quotations: Fostering Longitudinal Clinician–Patient Relationships and Building Trust**
Medical Oncology Progress Note	*As long as no dramatic change in symptoms and stable MRI would recommend continued observation. We discussed that we will slowly stays out MRIs over time and eventually he them as needed for new symptoms*
Medical Oncology Progress Note	*As he has been doing well for 2 years since completion of treatment, I think it is reasonable to start spacing out his visits and at some point we will likely go to as needed only*
Surgical Oncology Progress Note	*We could consider systemic treatment for control prior to another resection. If not, he can proceed with resection of tumor now and consider systemic therapy at a later date*
Response to Patient EHR Message	*But I do want you to understand that the consent form lists any possible risks to the treatment, Every chemotherapy we have given you also carries some risk to liver or organ failure. The risks are very minimal and I personally have not seen any issues with liver failure with this drug or class of drugs, but there will always be risks with any treatment you take*
Medical Oncology Progress Note	*I reviewed MRI results showing continued stability off of treatment. I discussed that I had even hoped for tumor shrinkage given the overlap in some active agents for both desmoid and lymphoma, but regardless there is no growth or symptoms*

Another patient expressed her interest in prioritizing pregnancy over immediate desmoid treatment as documentation from her medical oncologist states: “She and her husband are hoping to have children soon and she would like to make arrangements for desmoid treatment after this occurs, if possible.”

One patient with concerns regarding her employment status shared her reasoning for self‐discontinuation of desmoid‐directed treatment, which her medical oncologist summarizes:

She reports that she got a new job shortly after she started sorafenib and she was unable to return to clinic for labs and visits. She took the medication for 6 weeks and was found to be hypertensive on a work physical, which prompted her to stop taking the medication completely.

One patient had a treatment response with local therapy, but her employment status precluded her from repeat treatment as her medical oncologist reports:

[Patient] had cryoablation 18 months ago and does not want to have it again. Her pain became temporarily worse. She works as a waitress and is on her feet a majority of the time and this may cause her to miss many shifts.

At times, treatment decisions were made with considerations for reduced disruption to one's lifestyle surpassing the importance of potential treatment efficacy. A patient presented with such concerns and her medical oncologist documents:

I discussed with her that one of the most tolerable therapies is tamoxifen + sulindac [oral medications]. Although it has less of a chance of helping as some other options, its tolerability makes it appealing.

Patients with desmoid tumors also expressed a burden of psychosocial distress related to their disease course and therapy. One patient outlined these concerns to a social worker who summarizes their encounter:

[Her] mood is stressed, and she feels like a quitter because she has had to stop caring for her house and her kids. She feels defeated because this is the first time she's had to stop working due to her treatments. She has experienced setbacks in relationships with friends during difficult times, including desmoid treatments.

One patient described body image concerns to her medical oncologist who documented that:

Although she understands that surgical intervention could worsen the desmoid disease process, she is upset with the appearance of her hand, and desires consultation with the surgical team.

Another patient recalled her body image distress with prior lines of therapy and her medical oncologist notes this concern in their documented treatment plan:

We have discussed medication at lower doses, but she previously had bothersome hair and weight loss, and is nervous about re‐starting at all.

4 Fostering Longitudinal Clinician–Patient Relationships and Building Trust

Documentation referencing desmoid tumors and treatment plans emphasized the chronic nature of desmoid tumors (additional supporting quotations in Table 3). This was evident in a medical oncologist's documentation regarding a patient's treatment and surveillance options:

Should she have tumor recurrence, we would consider symptoms and potential risks with tumor growth and discuss if it made sense to initiate therapy at that time. Since she is young and will need serial imaging, I recommend MRI A/P for surveillance to limit risks of repeat contrast and radiation exposure.

Documentation also referenced anticipatory guidance for setting expectations for patients. One patient reported toxicities from therapy which guided adjustments made by her medical oncologist:

We advised that she decrease her dose to 400 mg daily to hopefully improve some of the side effects she is having, especially as treatment for this will be long‐term.

For some patients, anticipatory guidance was given in the form of contingency planning, outlining the next potential steps in treatment. At the conclusion of a visit, a medical oncologist detailed the spectrum of treatment possibilities for their patient based on planned imaging:

We will repeat an MRI of the chest wall in 4 months. If stable disease and no pain, we will increase the interval between surveillance scans. If pain due to the desmoid tumor and evidence of progression, we will discuss alternative systemic treatment options, including chemotherapy.

A patient expressed hesitancy in starting therapy, prompting his medical oncologist to offer tailored treatment recommendations:

We also discussed there can be more aggressive regimens in the future but if we start with less aggressive agents, we can rule in or out anti‐inflammatories as useful agents for him.

One patient shared her concerns about desmoid treatment effects on fertility and her medical oncologist documented the following in an attempt to reassure a patient's decision:

She shared that she and her husband are hoping to conceive soon, and she was worried about potential treatments impacting pregnancy. We discussed that we would not recommend holding off family planning in anticipation of need for treatment for her desmoid tumor, but we could consider repeating a few MRIs look to assess tumor growth if that would help reassure her decision.

## 4 Discussion

**FIGURE 2:**
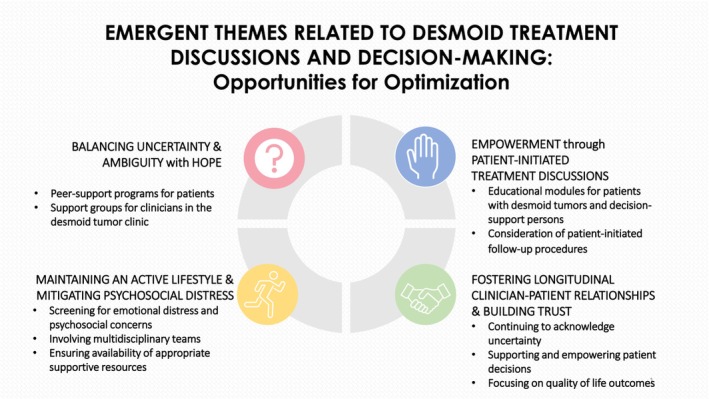
Opportunities for optimization.

This thematic analysis of the EHRs of patients with desmoid tumors and the discussions surrounding treatment and treatment decisions provides insight into this process in a real‐world clinical setting. While the identified themes of uncertainty and ambiguity, patient‐initiated treatment discussions, improvement in quality of life and distress mitigation, and the importance of longitudinal relationships and trust‐building are broad, their emergence in the space of desmoid tumor treatment discussions and decision‐making can help inform practical changes to improve these encounters (Figure 2).

Uncertainty and ambiguity were evident in the discussions of selection of treatment agent, timing of treatment, and the decision to proceed with surveillance. When confronting such unknowns, peer‐support programs specific to patients with desmoid tumors may be useful in providing tangible examples of outcomes in an ambiguous space and can foster the sense of hope that was also realized in our analysis.

Peer‐support programs for patients with cancer can improve satisfaction with medical care, personal relationships, and overall mood [22]. Some programs utilize online matching systems in which interested patients are matched with a volunteer with a similar disease history, and parties engage in phone calls or online messaging [23]. Given the rarity of desmoid tumors, it is reasonable to consider a similar program in this unique population where general concerns and the unavoidability of uncertainty will be shared. At present, large‐scale organizations such as The Desmoid Tumor Research Foundation (DTRF) and The Desmoid Project provide a repository of educational materials and support for patients, largely in the form of individualized, self‐led modules or webinars [24, 25]. Fostering a sense of community that offers meaningful interaction among patients through modules led by a clinician or by a “matching” program could provide an additional level of peer support on a smaller scale, perhaps even within a single institution where the likelihood of shared experience is even more probable.

Clinicians experienced similar challenges in coping with the lack of clarity surrounding desmoid tumor management, including the differentiation between desmoid‐related symptoms and other medical issues and recommending the appropriate timing of treatment. Such a lack of clarity contrasts with other cancer diagnoses in which well‐defined protocols with clear treatment indications often exist. Ongoing uncertainty surrounding desmoid tumors can affect the morale of clinicians.

Previous work has shown that peer support for clinicians can be effective in combatting this poor morale while enhancing empowerment, physical and mental health, and hope [26, 27]. Clinicians prefer to participate in peer relationships with colleagues who understand their professional responsibilities and associated stressors [28, 29, 30]. Clinicians providing care for patients with desmoid tumors share a unique set of experiences and, while formal peer support groups may not be needed, the baseline principles of community support can be adapted in less structured ways. This may include increased frequency of desmoid‐tumor specific national meetings or dedicated efforts to formulate a “desmoid‐tumor clinician list” comprised of clinicians interested in remaining in contact with others for networking purposes or for discussions regarding challenging patient scenarios.

In this analysis, many treatment discussions were initiated by patients or caregivers, and this did provide a sense of empowerment. Patients presented information from external resources to their health care providers or requested more information from their medical team to inform their treatment decisions. High‐quality medical decisions, defined as being both informed (accurate understanding of the options) and values‐concordant (consistent with the patient's underlying values) are necessary in true patient‐centered care [31, 32]. The initiative displayed by patients in this analysis suggests that patients with desmoid tumors seek high‐quality medical decisions as they sought both an intellectual understanding of options and a treatment outcome consistent with individual values.

The investigation into high‐quality decisions in cancer care has been robust. While patients with cancer in general desire shared decision‐making, one study including 192 participants noted that only half believed that choices were offered or that they were presented equally [33]. For patients with cancer, survival information is critical, and patients prefer this to be presented in simple, graphical displays [34]. Finally, one mixed‐methods study reported that the majority of participants felt that their clinicians struggled to weigh the pros and cons of cancer treatments, especially when quality and quantity of life were compared [35].

The distinction between a cancer diagnosis and a diagnosis of a desmoid tumor is important to note in the pursuit of a high‐quality decision. Clinicians caring for patients with desmoid tumors typically spend the majority of their clinical practice caring for patients with a cancer diagnosis where survival percentages and protocolized treatment choices are of increased importance. The current analysis shows that patients with desmoid tumors have different goals when assessing their treatment options, and to this end, clinicians are advised to preemptively provide the educational resources that patients desire. Providing such resources requires collaboration with national and international groups to create a variety of educational materials outlining the evolving treatment options available for desmoid tumors in a way that graphically depicts lifestyle effects of desmoid tumors and their directed therapies rather than survival statistics. As demonstrated in this analysis, family members often represent decision support persons (DSPs), parties who are engaged in patient decision‐making [36]. Clinicians may benefit from including DSPs in treatment discussions and decision‐making, and doing so may involve educational modules or decision aids to be viewed by both the patient and their DSP, in‐person, or virtually over geographical distance.

Finally, clinicians in the desmoid treatment space were supportive of patient‐initiated treatment discussions and decisions, extending into the surveillance setting. One model utilized in some cancer survivorship settings is known as patient‐initiated follow‐up (PIFU) [37, 38]. In this model, patients trigger follow‐up visits or imaging according to individual needs rather than relying on inflexible scheduling. PIFU may be a reasonable consideration for many patients with stable desmoid tumors given their nonmalignant nature and may serve to reduce unnecessary contact with the health care system while also furthering patient empowerment and autonomy [39, 40].

Previous work has demonstrated the high prevalence of emotional and psychosocial distress in patients with desmoid tumors [5, 7]. In this analysis, discussions surrounding treatment options often focused on mitigating those potential outcomes while maintaining an active lifestyle. To better support these discussions, a more complete assessment of overall psychosocial health at the time of diagnosis of a desmoid tumor may help elucidate any pre‐existing concerns. Importantly, repeating this assessment throughout the disease trajectory will allow the clinician to tailor their approach to treatment recommendations.

Teams providing medical care to patients with desmoid tumors are generally comprised of medical, surgical, and perhaps radiation oncologists, along with their respective nursing support. Incorporating mental health providers, such as a social worker, into these teams may serve as an additional layer of support for patients to communicate concerns related to impaired quality of life due to their tumor or treatment. If additional supportive care is required, it is vital that the resources available meet the needs of patients with desmoid tumors. As most patients with desmoid tumors receive care at large cancer centers, it may be that the associated resources are more appropriate for patients with malignant diagnoses. An internal review of those services and education for those providers may be needed to optimize their efficacy for patients with desmoid tumors.

The identified theme of “longitudinal relationships and building trust” can be seen as one encompassing the three themes previously described. Addressing with transparency the uncertainty and ambiguity of desmoid tumors and treatment efficacy, empowering patient‐initiated treatment discussions and decisions and striving to improve quality of life and mitigate distress all foster a healthy clinician–patient relationship. For patients with desmoid tumors, these relationships may last for many years and evolve along with the trajectory of the disease entity itself. Oncologist in general can influence their patients' trust by enhanced conveyance of their competence, honesty and caring [41]. This analysis has generated suggestions to foster these important qualities in an effort to further improve the communication surrounding desmoid tumor treatment discussions and decision making.

## 5 Implications

The clinical implications of the emergent themes surrounding discussions of desmoid tumor treatment and treatment decision making stem from the identification of opportunities for optimization of this process. These opportunities include establishment of local, community‐based peer‐support programs for patients and clinicians, educational modules for patients and DSPs, consideration of patient‐initiated follow‐up, systematic screening for emotional distress, and ensuring adequate access to resources to address the psychosocial needs of patients with desmoid tumors. These clinical implications also inform the research efforts needed to effectively carry out these suggestions.

## 6 Limitations

This study allows for an appreciation of the longitudinal nature of desmoid tumors as documentation at different time points in the disease course was analyzed. Limitations include the academic single‐center nature of this study as approaches to treatment discussions vary across types of institutions. All patients with desmoid tumors were included, both those with FAP‐related tumors and those with idiopathic tumors. Patients with FAP may have different perspectives on the presented themes based on their expected disease trajectory, and a separate future analysis may explore this further. This analysis took place during a time in which new therapies emerged for desmoid tumors, and some of the encounters utilized for analysis consisted of clinical trial documentation, often more rigorous and prescriptive than non‐trial documentation. The methodology of this study also presents limitations as not all aspects of a clinical encounter are represented in EHRs, most notably the direct perspective of patients and families and attached emotions. As noted previously, of the 195 total abstracted passages of documentation, only 6 (approximately 4%) represented patient‐initiated messages. As this study was a reflexive thematic analysis performed by cancer clinicians, that perspective shapes the interpretation of themes and their potential implications for practice. Reflexive thematic analysis values that subjectivity as a strength when it is acknowledged, and accordingly, we note that here. Utilizing EHR documentation as source material for qualitative research in general is uncommon, and this study highlights the unique possibilities as well as limitations of this methodology. For example, patients with desmoid tumors can struggle with self‐identity and garnering support networks given the nonmalignant nature of their disease [5]. However, this was not evident in our analysis of 195 passages of documentation, suggesting that the medical record indeed underestimates the psychosocial challenges of patients with desmoid tumors. Future research would be strengthened by presenting the emergent themes of this study to patients living with desmoid tumors to assess their resonance and incorporating in‐depth interviews with patients to advance knowledge of the unique needs of this population.

There is often additional time spent discussing treatment and counseling patients that is not captured in written documentation. Nevertheless, inductive qualitative analysis of medical records is an insightful yet underused methodology, making the contributions of the current study unique across psycho‐oncology research generally, and meaningful within the desmoid tumor knowledge base specifically.

## 7 Conclusions

This qualitative thematic analysis of the EHRs of patients with desmoid tumors and the discussions surrounding treatment and treatment decisions underscores the unique nature of both desmoid tumors and the challenges that patients with desmoid tumors may face when considering treatment, especially when compared to individuals with malignant tumor diagnoses. The themes identified in this study suggest that there are opportunities to improve the communication between clinicians and patients when discussing desmoid tumor treatments and provide a foundation for further investigation into interventions to support informed decision‐making.

## Author Contributions

**Victoria Wytiaz:** conceptualization (lead), formal analysis (lead), methodology (lead), writing – original draft (lead). **Tianyi Wang:** formal analysis (equal), writing – review and editing (equal). **Scott Schuetze:** data curation (supporting), writing – review and editing (supporting). **Nina J. Francis‐Levin:** methodology (supporting), writing – review and editing (supporting). **Rashmi Chugh:** data curation (supporting), writing – review and editing (supporting).

## Conflicts of Interest

The authors declare no conflicts of interest.

## Data Availability

The data underlying this article will be shared on reasonable request to the corresponding author.
